# The noncatalytic regions of the tyrosine kinase Tnk1 are important for activity and substrate specificity

**DOI:** 10.1016/j.jbc.2022.102664

**Published:** 2022-11-02

**Authors:** Sultan Ahmed, W. Todd Miller

**Affiliations:** 1 Department of Physiology and Biophysics, Stony Brook University, Stony Brook, New York, USA; 2 Department of Veterans Affairs Medical Center, Northport, New York, USA

**Keywords:** nonreceptor tyrosine kinase, ATP, phosphorylation, enzyme purification, enzyme kinetics, Ack family, Tnk1, EGFR, epidermal growth factor receptor, FBS, fetal bovine serum, NRTK, nonreceptor tyrosine kinase, SAM, sterile alpha motif, Tnk1, thirty-eight negative kinase 1, UBA, ubiquitin association domain, WASP, Wiskott-Aldrich Syndrome Protein

## Abstract

Human Tnk1 (thirty-eight negative kinase 1) is a member of the Ack family of nonreceptor tyrosine kinases. Tnk1 contains a sterile alpha motif, a tyrosine kinase catalytic domain, an SH3 (Src homology 3) domain, and a large C-terminal region that contains a ubiquitin association domain. However, specific physiological roles for Tnk1 have not been characterized in depth. Here, we expressed and purified Tnk1 from Sf9 insect cells and established an *in vitro* assay system using a peptide substrate derived from the Wiskott-Aldrich Syndrome Protein (WASP). By Tnk1 expression in mammalian cells, we found that the N-terminal SAM domain is important for self-association and kinase activity. We also studied a fusion protein, originally discovered in a Hodgkin’s Lymphoma cell line, that contains an unrelated sequence from the C17ORF61 gene fused to the C-terminus of Tnk1. Cells expressing the fusion protein showed increased tyrosine phosphorylation of cellular substrates relative to cells expressing WT Tnk1. A truncated Tnk1 construct (residues 1–465) also showed enhanced phosphorylation, indicating that the C17ORF61 sequence was dispensable for the effect. Additionally, *in vitro* kinase assays with the WASP peptide substrate showed no increase in intrinsic Tnk1 activity in C-terminally truncated constructs, suggesting that the truncations did not simply remove an autoinhibitory element. Fluorescence microscopy experiments demonstrated that the C-terminus of Tnk1 plays an important role in the subcellular localization of the kinase. Taken together, our data suggest that the noncatalytic regions of Tnk1 play important roles in governing activity and substrate phosphorylation.

Tnk1 (Thirty-eight negative Kinase 1) is a nonreceptor tyrosine kinase (NRTK) that was first identified in CD34+/Lin-/CD38-hematopoietic progenitor cells (1). Tnk1 is widely expressed in fetal tissues, while in healthy adults, expression is strongest in the prostate, testis, ovary, small intestine, and colon (2). Tnk1 is a member of the Ack family of NRTKs, a group that in humans contains only Tnk1 and Ack1 (activated Cdc42-associated kinase, also called Tnk2). From the N-terminus to C-terminus, Tnk1 consists of a SAM (sterile alpha motif) domain, a kinase catalytic domain, an SH3 domain, and a proline-rich C-terminal tail whose function has not been fully elucidated (Fig. 1). The C-terminus of Ack1 contains additional regions that are lacking in Tnk1: a Cdc42-binding CRIB domain, a clathrin interaction motif, and a Mig6-homology region (Fig. 1) (3, 4, 5, 6). The C-terminus of Ack1 possesses a ubiquitin-association domain (UBA), and a UBA has been recently identified in Tnk1 (Fig. 1) (7). These portions of Ack1 play important roles in enzyme regulation and in mediating protein–protein interactions (8).

**Figure 1:**
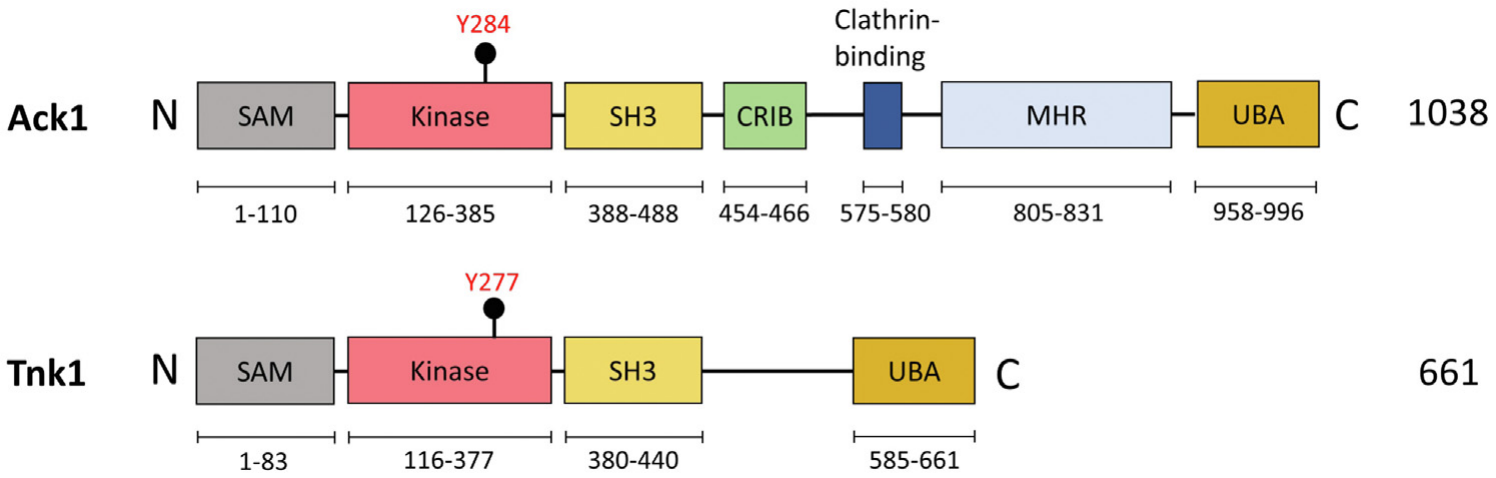
**Ack family homology.** The domain architectures of Ack1 and Tnk1. Y284 of Ack1 is the major autophosphorylation site within the activation loop (18), and Y277 of Tnk1 is the corresponding residue. The range of residues for the Tnk1 SAM domain is based on the predicted structure from the AlphaFold database (33, 34). CRIB, Cdc42/Rac-interactive domain; MHR, Mig6-homology region; SAM, sterile alpha motif; Tnk1, thirty-eight negative kinase 1; UBA, ubiquitin-binding domain.

The normal physiological functions of Tnk1, and its potential involvement in disease, are not well understood. Genome-wide association studies of Alzheimer’s disease have suggested a linkage with Tnk1 (9). A possible mechanism involves the ability of Tnk1 to induce TNFα-dependent apoptosis by preventing activation of NF-κB (2), as TNFα is known to be important in driving the progression of Alzheimer’s disease (10). In cancer, Tnk1 may act as a tumor suppressor or as an oncogene, depending on cell context. Overexpression of Kos1 (the murine homolog of Tnk1) in Ras (G12V)-driven lung cancer cells inhibited cell growth (11). Knockout of Kos1 resulted in the spontaneous formation of lymphomas and carcinomas (12). The proposed mechanism for this tumor suppressive function involves downregulation of the Ras/Raf/MAPK pathway by Tnk1 phosphorylation of Grb2 (13). On the other hand, RNAi kinome screens have demonstrated that Tnk1 plays a role in the viability of the immortalized pancreatic cell lines BxPC3 and MiaPaCa-2 (14) and in *ex vivo* cultures of samples from patients with acute myeloid leukemia and B-cell and T-cell acute lymphoblastic leukemia (7). A correlation was also observed between high Tnk1 expression and decreased survival of acute lymphoblastic leukemia patients. A unique form of Tnk1 was discovered in the Hodgkin’s Lymphoma cell line L540: the 5′ portion of the Tnk1 gene (encoding the kinase domain) was fused to sequences from the C17ORF61 (chromosome 17 open reading frame 61) gene. This variant of Tnk1 (Tnk1^Fusion^) promoted cellular growth and phosphorylation of proliferative substrates (15).

To facilitate studies of Tnk1 activity and regulation, we report here the expression and purification of the enzyme from Sf9 insect cells. We developed an *in vitro* assay based on phosphorylation of a peptide derived from the Wiskott-Aldrich Syndrome Protein (WASP). Using this assay, we compared the activities of WT Tnk1, N- and C-terminal deletion constructs, and Tnk1^Fusion^. These results suggest functional roles for the noncatalytic N- and C-termini of Tnk1 and point to a potential mechanism for the pro-proliferative phenotype elicited by the Tnk1^Fusion^ mutant in Hodgkin’s Lymphoma cells.

## Results

### Purification and biochemical characterization of Tnk1

**Figure 2:**
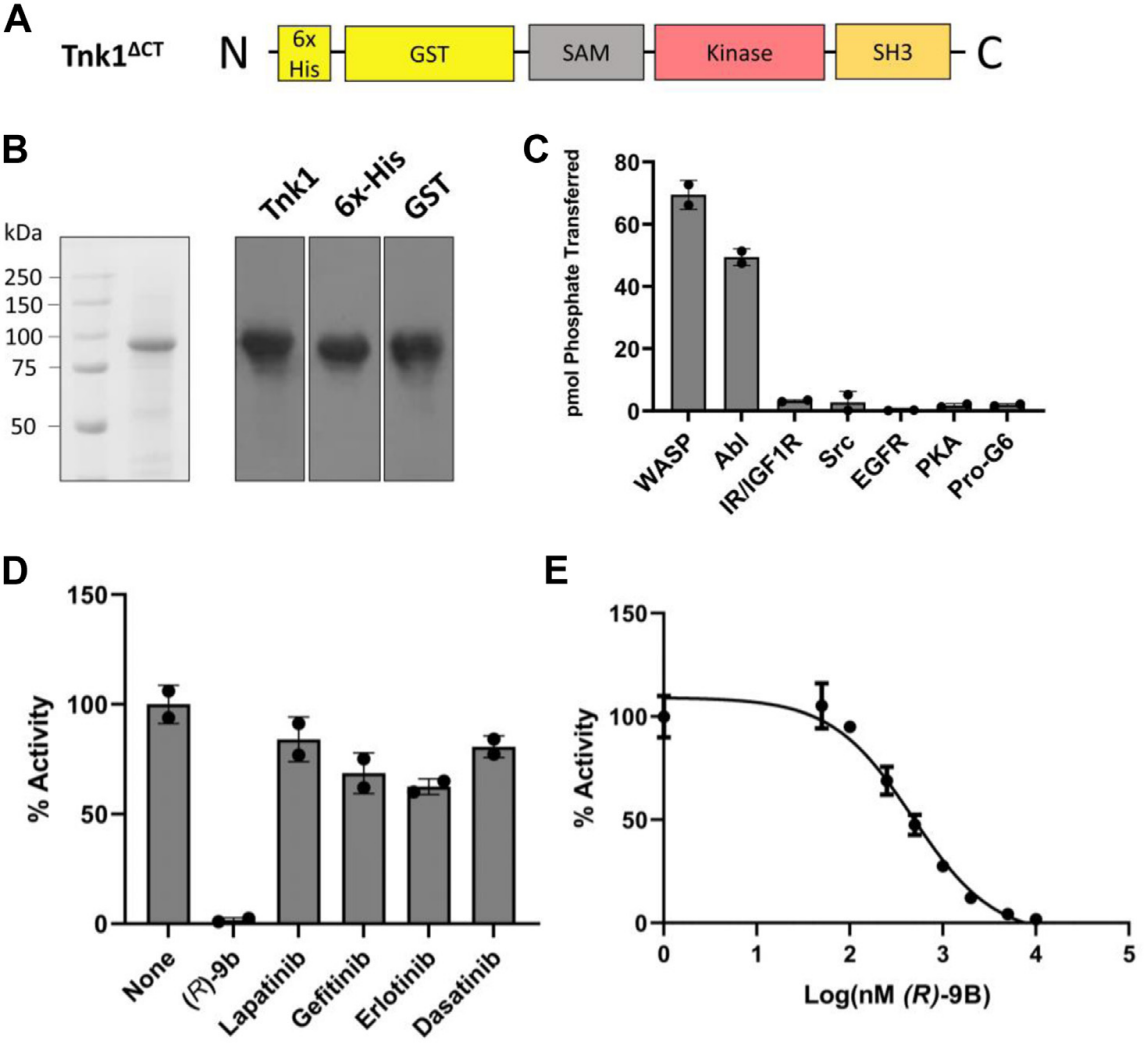
***In vitro* activity of Tnk1**^**ΔCT**^**.***A*, the Tnk1^ΔCT^ construct expressed and purified from Sf9 cells with N-terminal 6x-His and GST tags. *B*, following Ni-NTA affinity chromatography, Tnk1^ΔCT^ was analyzed by SDS-PAGE with Coomassie staining and by Western blotting using the indicated antibodies. *C*, purified Tnk1^ΔCT^ was assayed *in vitro* using γ^32^-ATP and a series of synthetic peptides at 1 mM concentrations. *D*, the activity of Tnk1^ΔCT^ was measured in the presence of the indicated inhibitors (10 μM) using 1 mM WASP peptide. *E*, the activity of Tnk1^ΔCT^ was measured in the presence of varying concentrations of *(R)*-9b. Percent activity was calculated relative to a DMSO control. *In vitro* assays were performed at 200 nM enzyme and 1 mM ATP concentrations. Error bars show SD. DMSO, dimethyl sulfoxide; Tnk1, thirty-eight negative kinase 1; WASP, Wiskott-Aldrich Syndrome Protein.

There is no reported biochemical characterization of purified Tnk1 in the literature. Initial attempts at expression in *Escherichia coli* were unsuccessful, either in the presence or absence of GroEL/GroES chaperones and YopH phosphatase (16), due to the accumulation of Tnk1 in the insoluble pellet after centrifugation of cell lysates. To produce Tnk1, we generated a recombinant baculovirus encoding a C-terminally truncated construct (Tnk1^ΔCT^: 1–510aa) with the SAM, kinase, and SH3 domains intact. We engineered this 87 kDa construct to contain an N-terminal 6x-His tag for affinity chromatography followed by a GST tag to improve the solubility of the protein (Fig. 2*A*). We expressed and purified Tnk1^ΔCT^ in *Spodoptera frugiperda* (Sf9) insect cells using Ni-NTA chromatography and confirmed the identity of the protein by Western blotting (Fig. 2*B*). We used this GST-tagged protein for subsequent activity measurements, because we found that cleavage of the GST tag resulted in protein that was not monodisperse when analyzed by-size exclusion chromatography.

We first established an *in vitro* assay for purified Tnk1 using [^32^P-ATP] and the phosphocellulose paper-binding assay (17) and studied the peptide substrate specificity of the enzyme. We screened synthetic peptides containing the substrate recognition sequences of several representative tyrosine kinases (Abl, IR/IGF1R, Src, and epidermal growth factor receptor (EGFR)) (18, 19). We included a substrate for PKA, as Ack1 possesses detectable Ser/Thr kinase activity (20). We also included a synthetic peptide modeled on the Src/Ack1 phosphorylation site (Y256) of WASP; we previously showed that this peptide is phosphorylated by Ack1 with more favorable kinetics than any other reported substrate (21). Finally, we tested a peptide (Pro-G6) that combines a phosphorylation site with an SH3-binding sequence. This peptide shows enhanced phosphorylation by NRTKs (e.g., Src) whose SH3 domains act as substrate targeting motifs (22). Tnk1 activity was highest against the WASP peptide (Fig. 2*C*). The Abl peptide also exhibited a significant level of phosphorylation, suggesting potential overlap of the substrate specificity between Tnk1 and Abl. This may be due to common specificity-determining residues in the catalytic domains of the two enzymes (Fig. S1). Phosphorylation of the other peptides was low or undetectable (Fig. 2*C*). Next, we carried out steady-state kinetic experiments with the WASP peptide (Fig. S2). These experiments gave a K_m (ATP)_ value of 70 μM and a K_m (peptide)_ value of 214 μM (Fig. S2), indicating that the WASP peptide is a good substrate for measuring *in vitro* Tnk1 activity.

We employed the *in vitro* assay with WASP peptide to test the effectiveness of a series of FDA-approved tyrosine kinase inhibitors against Tnk1. We tested the EGFR family kinase inhibitors lapatinib, erlotinib, and gefitinib, as well as the Src/Abl family inhibitor dasatinib. We included compound *(R)*-9b, an ATP-competitive small molecule inhibitor of Ack1 that is highly effective *in vitro* and *in vivo* (23). At a single concentration of 10 μM, erlotinib and gefitinib gave 30 to 40% inhibition; the other FDA-approved inhibitors were somewhat less effective (Fig. 2*D*). *(R)*-9b completely inhibited Tnk1 (Fig. 2*D*). Dose-response experiments showed that under our assay conditions, the IC_50_ of *(R)*-9b was 470 nM (Fig. 2*E*). These results from our inhibitor experiments suggest that the experimental Ack1 inhibitor *(R)*-9b would also be useful as a tool for probing Tnk1 function.

### Role of the SAM domain of Tnk1

**Figure 3:**
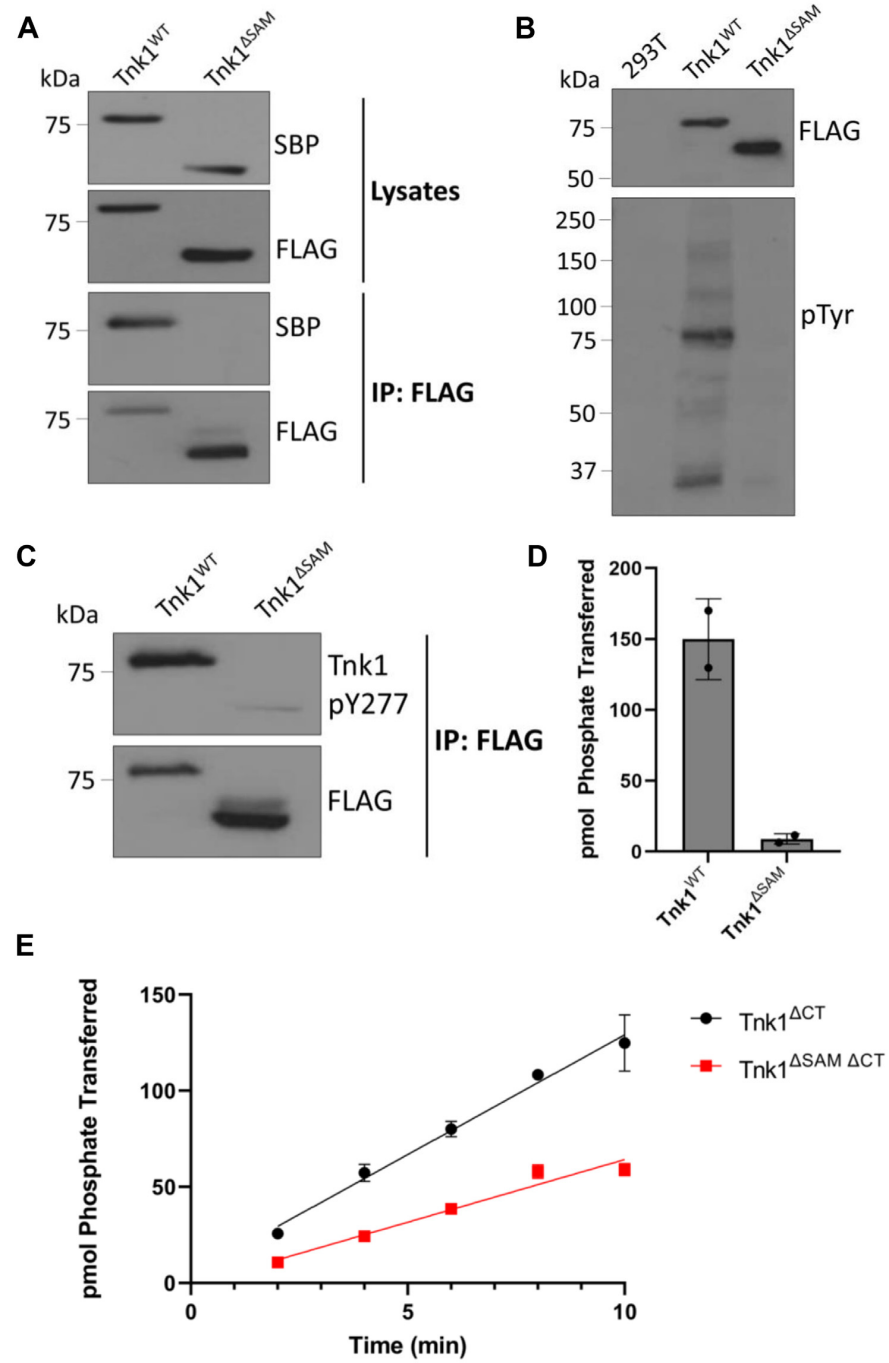
**Self-association of Tnk1 through its N-terminal SAM domain.***A*, *top*: lysates of HEK293T cells coexpressing FLAG-tagged and SBP-tagged forms of the indicated constructs were analyzed by Western blotting with FLAG and SBP antibodies. Bottom: the lysates were subjected to immunoprecipitation with anti-FLAG resin. The anti-FLAG precipitates were analyzed by Western blotting. An expanded view of these gels, including analyses of cells expressing the individual tagged enzymes, is shown in Fig. S3. *B*, lysates of cells expressing FLAG-tagged Tnk1^WT^ and Tnk1^ΔSAM^ were analyzed by anti-FLAG and anti-pTyr Western blotting. *C*, anti-FLAG immunoprecipitates of cells expressing Tnk1^WT^ and Tnk1^ΔSAM^ were analyzed by Western blotting with phospho-Tnk1 (pY277) antibody. *D*, *in vitro* activity assay using anti-FLAG immunoprecipitates from cells expressing Tnk1^WT^ and Tnk1^ΔSAM^. Reactions were carried out with [^32^P-ATP], using 1 mM ATP and 1 mM WASP peptide. *E*, *in vitro* activity assay of purified Tnk1^ΔCT^ and Tnk1^ΔSAMΔCT^. Reactions contained γ^32^-ATP, 400 μM ATP, 1 mM WASP peptide, and 100 nM enzyme and were analyzed using the phosphocellulose-binding assay. Error bars show SD. SAM, sterile alpha motif; Tnk1, thirty-eight negative kinase 1; WASP, Wiskott-Aldrich Syndrome Protein.

The N-terminal SAM domain of Ack1 has been shown to facilitate the self-association and activation of the enzyme (24). To determine whether the SAM domain of Tnk1 plays a similar role, we generated plasmids encoding FLAG-tagged and SBP-tagged Tnk1 (WT and SAM-deleted) for expression in mammalian cells. The proteins were all expressed in HEK293T cells (Figs. 3*A*, top and S3). We analyzed Tnk1 self-association by anti-FLAG co-immunoprecipitation. When expressed alone, neither SBP-tagged Tnk1^WT^ nor SBP-tagged Tnk1^ΔSAM^ was immunoprecipitated with FLAG (Fig. S3). SBP-tagged Tnk1^WT^ coimmunoprecipitated with FLAG-tagged Tnk1 (Fig. 3*A*, bottom). Deletion of the SAM domain led to a significant reduction in the amount of SBP-tagged Tnk1^ΔSAM^ that coimmunoprecipitated with FLAG-tagged Tnk1^ΔSAM^ (Fig. 3*A*, bottom). To determine whether the SAM domain is important for Tnk1 activity, we analyzed the levels of phosphotyrosine-containing proteins in lysates of transfected HEK 293T cells and found a significant decrease in Tnk1 activity (Fig. 3*B*). We found a similar reduction in the levels of phosphorylation of Tyr277, which corresponds to the predicted major site of autophosphorylation within the activation loop of Tnk1 (Fig. 3*C*). To compare activities directly, we immunoprecipitated FLAG-tagged Tnk1 (WT and Tnk1^ΔSAM^) and measured *in vitro* activities using [^32^P]-ATP and the WASP peptide. These experiments showed that removal of the SAM domain caused a large reduction in Tnk1 activity (Fig. 3*D*). Next, we generated a recombinant baculovirus encoding the C-terminally truncated construct (Tnk1^ΔCT^; Fig. 2*A*) but lacking the SAM domain and the linker between SAM and kinase domains (Tnk1^ΔSAMΔCT^). We expressed and purified this protein in Sf9 insect cells. Activity measurements at saturating concentrations of ATP and peptide showed a ∼2-fold decrease in the rate for the Tnk1 construct lacking the SAM domain (Fig. 3*E*). Together, these results suggest that the SAM domain is important for the cellular self-association and activation of Tnk1, in a similar manner as was previously observed for Ack1.

### Role of the C-terminus of Tnk1

**Figure 4:**
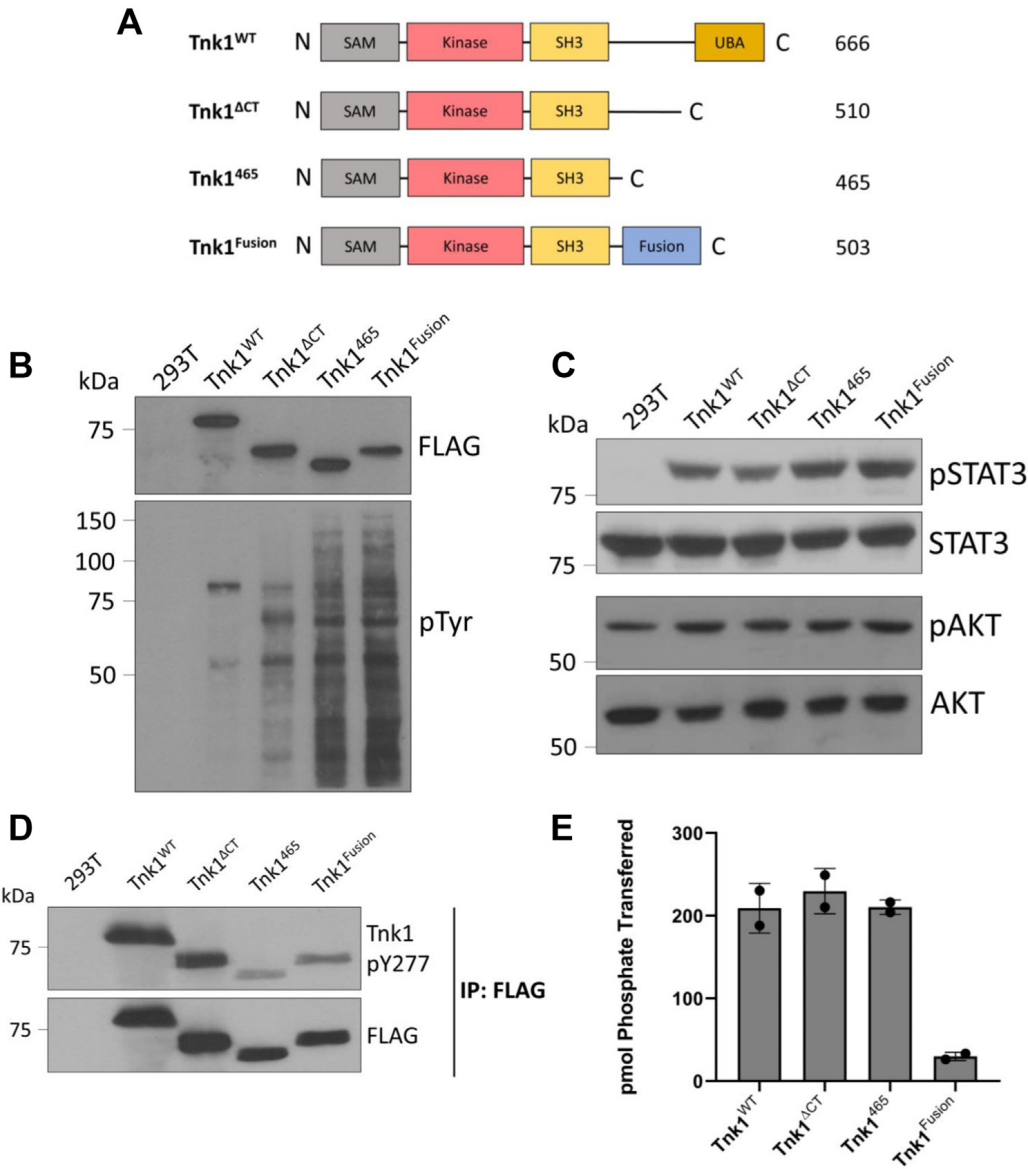
**Expression and activity of Tnk1 mutants in HEK 293T cells.***A*, mammalian expression constructs encoding Tnk1^WT^, Tnk1^Fusion^, and two C-terminal truncations. The constructs contain N-terminal FLAG tags. *B*, lysates from HEK293T cells expressing Tnk1^WT^ and mutants were analyzed by anti-FLAG and anti-pTyr Western blotting. *C*, Western blotting with the indicated antibodies was performed to analyze phosphorylation of downstream signaling partners of Tnk1. *D*, lysates were analyzed by Western blotting with anti-phospho-Tnk1 (pY277) antibody. *E*, *in vitro* activity assay of Tnk1 (WT and mutants) immunoprecipitated from HEK293T cell lysates. Reactions were carried out using 1 mM ATP and 1 mM WASP peptide. Error bars show SD. Tnk1, thirty-eight negative kinase 1; WASP, Wiskott-Aldrich Syndrome Protein.

A Tnk1 variant with a truncated C-terminus fused to a 39-residue segment from C17ORF61 (Tnk1^Fusion^) showed increased activity (relative to WT Tnk1) in a Hodgkin’s Lymphoma cell line (15). As suggested by Gu *et al.*, truncation of the C-terminus of Tnk1 in Tnk1^Fusion^ might remove an autoinhibitory element, activating the kinase. Additionally or alternatively, the fusion sequence itself might impart higher activity to Tnk1. Based on recent studies by Chan *et al.* (7), we also considered the possibility that alterations in the Tnk1 C-terminus could affect substrate phosphorylation indirectly, through changes in subcellular localization. To test these possibilities, we produced mammalian cell expression constructs for WT Tnk1 and a series of C-terminal variants (Fig. 4*A*). Tnk1^Fusion^ is identical to the activated protein found in Hodgkin’s Lymphoma cells. Tnk1^465^ corresponds to Tnk1^Fusion^ protein minus the 39-residue fusion sequence, allowing us to test the effect of the C17ORF61 segment on activity. The Tnk1^ΔCT^ construct consists of residues 1 to 510 and corresponds to the protein we purified from insect cells for biochemical studies. When transiently transfected into HEK293T cells, these FLAG-tagged Tnk1 proteins were expressed at similar levels (Fig. 4*B*). We analyzed global phospho-tyrosine levels in HEK293T cell lysates by Western blotting with anti-pTyr antibody. Although Tnk1^WT^ and Tnk1^ΔCT^ showed comparable levels of cellular phosphotyrosine-containing proteins, there was a marked increase in the phosphorylation of proteins in cells transfected with Tnk1^465^ and Tnk1^Fusion^ (Fig. 4*B*). These results suggested that truncation of Tnk1 to amino acid 465, but not to 510, results in a hyperactivated kinase. The activities of Tnk1^465^ and Tnk1^Fusion^ were similar in this assay, arguing against a positive role for the 39-residue fusion segment. All of these C-terminal deletions had higher activity than Tnk1^ΔSAM^ (Fig. S4).

Next, we examined phosphorylation of the Tnk1 downstream signaling partners STAT3 and AKT (15). Using cell lysates of transfected HEK293T cells, we carried out Western blotting with the activation state-specific antibodies STAT3 (pY705) and AKT (S473) (25, 26). Expression of Tnk1^465^ and Tnk1^Fusion^ modestly increased the phosphorylation of pSTAT3 but not pAKT (Fig. 4*C*). It is not currently known whether STAT3 and AKT serve as direct substrates for Tnk1 or alternatively if other Tnk1 substrates lead to phosphorylation of STAT3 and AKT. In any case, these results raised the possibility that deletion of the Tnk1 C-terminus increases the phosphorylation of some, but not all, cellular proteins.

To compare the activities of the Tnk1 constructs, we first probed for the autophosphorylation site at pY277 in the predicted activation loop of the Tnk1 catalytic domain. Phosphorylation at this site and at Y287 is required for activity (13). We carried out anti-FLAG immunoprecipitation reactions and measured Y277 phosphorylation with a phosphospecific antibody. Unexpectedly, the two shorter constructs (Tnk1^465^ and Tnk1^Fusion^) showed lower levels of Y277 phosphorylation than WT Tnk1 (Fig. 4*D*), in contrast to their apparent higher activity in Figure 4*B.* A caveat is that phosphorylation at Y277 is an indirect measure of Tnk1 activity; Y277 might be autophosphorylated or serve as a phosphorylation site for another HEK293T kinase. Furthermore, we do not know the phosphorylation status of Y287 in these constructs. To assess Tnk1 activity directly, we immunoprecipitated the constructs and carried out *in vitro* activity assays using [^32^P-ATP] and the synthetic WASP peptide. There was no change in activity between Tnk1^WT^, Tnk1^ΔCT^, and Tnk1^465^, while Tnk1^Fusion^ exhibited reduced activity (Fig. 4*E*). We confirmed the lower activity of the fusion protein using a different peptide substrate (Fig. S5). While the reason for the low *in vitro* kinase activity of Tnk1^Fusion^ is currently unclear (see Discussion), the results for Tnk1^465^ suggest that the increased levels of cellular pTyr (Fig. 4*B*) do not arise from the deletion of an autoinhibitory region. Instead, we hypothesized that the increased cellular pTyr levels for Tnk1^465^ and Tnk1^Fusion^ were due to changes in binding and/or phosphorylation of particular cellular substrates.

**Figure 5:**
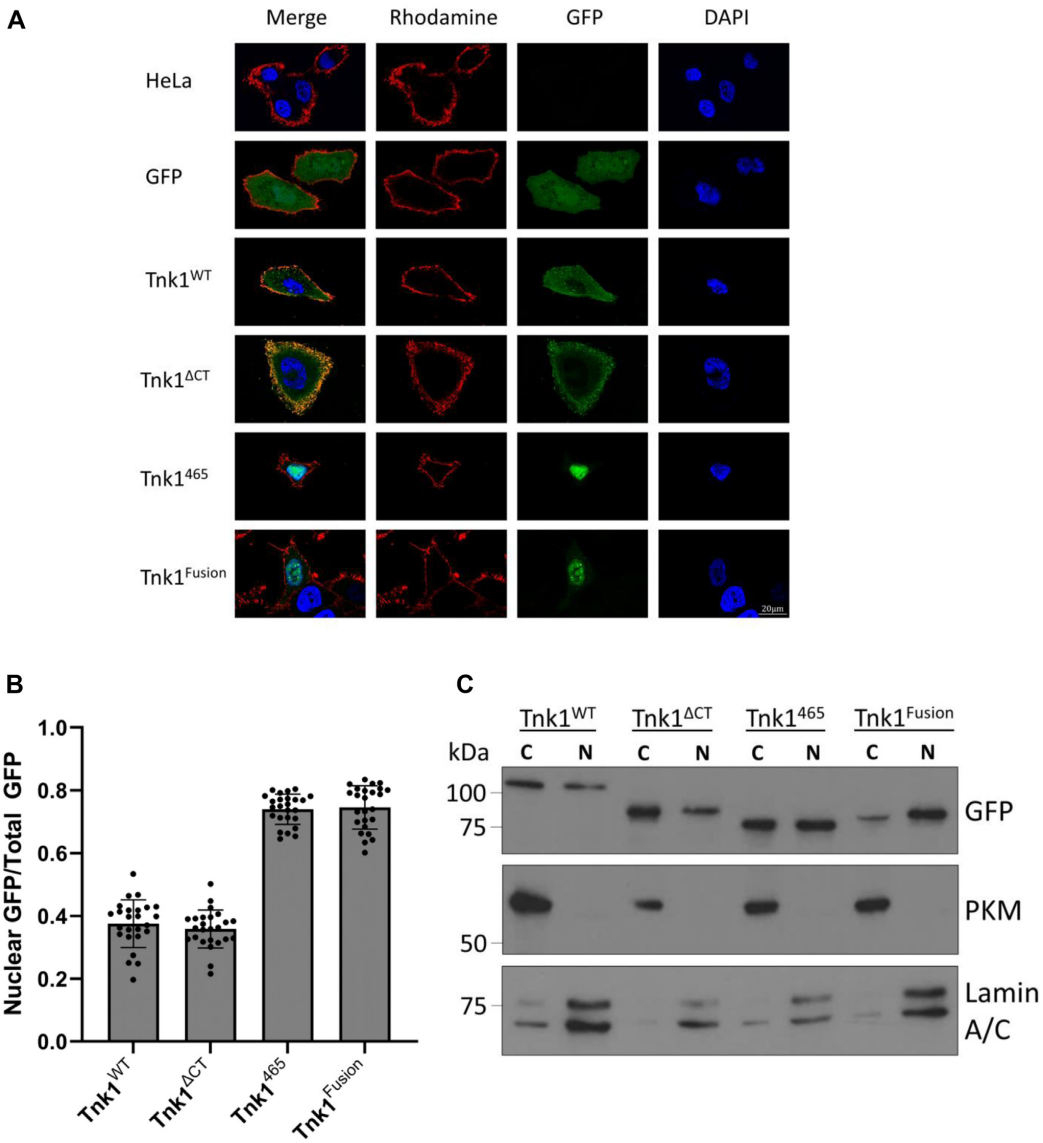
**Subcellular localization of Tnk1 proteins.***A*, HeLa cells were transiently transfected with WT and mutant forms of Tnk1 containing an N-terminal GFP tag and analyzed by fluorescence microscopy. WGA-rhodamine (wheat germ agglutinin) in *red* was used as a membrane marker. GFP in *green* was used to observe localization of Tnk1. DAPI in *blue* was used as a nuclear marker. The scale bar represents 20 μM. *B*, Tnk1 localization was quantified by taking the ratio of nuclear GFP signal to total GFP signal (nuclear + cytoplasmic) from panel A using an average of 25 cells per protein. Error bars show SD. *C*, subcellular fractionation of transiently transfected HeLa cells was performed to isolate the cytoplasmic and nuclear fractions. Anti-GFP was used to detect levels of GFP-tagged Tnk1. PKM (Protein Kinase M) was used as a cytoplasmic marker. Lamin A/C was used as a nuclear marker. Tnk1, thirty-eight negative kinase 1.

A potential mechanism for a change in substrate specificity could be an alteration in the subcellular localization of the truncated Tnk1 proteins. To investigate this possibility, we generated GFP-tagged constructs encoding Tnk1^WT^, Tnk1^ΔCT^, Tnk1^465^, and Tnk1^Fusion^ and carried out fluorescence microscopy studies. In our initial experiments with HEK293T cells, we found that the transfected cells were not sufficiently adherent to permit quantitation. Consequently, we carried out these experiments in HeLa cells (Fig. 5*A*). We confirmed that expression of the GFP-tagged constructs in HeLa cells gave similar results to those obtained in HEK293T cells by anti-pTyr Western blotting of cell lysates (Fig. S6). To quantify the microscopy results, we analyzed the ratio of nuclear GFP signal to the total GFP signal in both the cytosol and nucleus. We found an approximate two-fold increase in nuclear GFP for Tnk1^465^ and Tnk1^Fusion^ as compared to Tnk1^WT^ and Tnk1^ΔCT^ (Fig. 5*B*). Interestingly, Tnk1^Fusion^ formed puncta that were uniformly dispersed throughout the whole cell. To corroborate the microscopy results, we performed cellular fractionation of transfected HeLa cells and probed for anti-GFP in the cytosolic and nuclear fractions (Fig. 5*C*). Levels of Tnk1^WT^ and Tnk1^ΔCT^ were higher in the cytoplasmic fraction than the nuclear fraction. Tnk1^465^ had a roughly equal distribution, while Tnk1^Fusion^ showed an increased proportion of nuclear signal relative to cytosolic signal. Our interpretation of these results is that truncation of the C-terminus of Tnk1 causes a mislocalization of the enzyme, leading to increased phosphorylation of cellular proteins without a direct effect on the enzyme’s intrinsic kinase activity.

## Discussion

In this paper, we have established a method of expressing and purifying active Tnk1, and we identified an efficient peptide substrate based on the WASP protein. This assay system should facilitate further studies into the activity of Tnk1 and aid in the search for proteins and small molecules that regulate activity. We used this assay system to measure the activities of truncated forms of Tnk1 expressed in mammalian cells. Although expression of Tnk1^465^ and Tnk1^Fusion^ gave an increase in cellular pTyr (relative to WT Tnk1), there was no increase in intrinsic kinase activity, arguing against the presence of an autoinhibitory motif. Instead, our results suggest that the C-terminus of Tnk1 contains a sequence for cytosolic localization and that the altered activity of Tnk1^465^ and Tnk1^Fusion^ is due to their entry into the nucleus. Our findings with Tnk1^Fusion^ indicate that the 39-residue fusion sequence does not make a direct contribution to activity. It is also conceivable that the fusion sequence promotes Tnk1 aggregation, since *in vitro* activity was very low (Fig. 4*E*) and this protein showed a tendency to form puncta in HeLa cells (Fig. 5*A*). Our results are consistent with a recent study showing that phosphorylation of S502 in the C-terminus of Tnk1 leads to 14-3-3 binding and cytosolic localization (7). It is unclear whether the nuclear entry of Tnk1^Fusion^ is due to the presence of a nuclear localization signal in the protein sequence or the association with a nuclear entry chaperone.

The functional importance of the other domains of Tnk1 are not completely understood. The presence of an N-terminal SAM domain is a unique feature of the Ack family of NRTKs. SAM domains are small helical modules that mediate homo- and hetero-oligomerization (27). The SAM domain of Ack1 is required for its self-association and promotes kinase activation by autophosphorylation (24). Tnk1 contains a predicted SAM domain (Fig. 1). Our results suggest that like Ack1, the N-terminal SAM domain of Tnk1 is important for self-association and for activity in cells and *in vitro* (Fig. 3). We hypothesize that Tnk1 requires intermolecular autophosphorylation at Y277 for full activity and that the reduced activity of Tnk1^ΔSAM^ (Fig. 3) stems from a reduction in the efficiency of this process.

In contrast to Ack1, Tnk1 lacks a CRIB domain and is presumably not subject to the Cdc42-dependent activation observed for Ack1 (20, 28). Binding of the UBA domain in the C-terminus of Tnk1 to ubiquitin stabilizes an active form of the enzyme (7). Similarly, the ubiquitination of Ack1 through its UBA is required for its degradation. The UBA domain of Ack1 also facilitates the degradation of EGFR following EGF treatment (29). A cancer-associated mutation in the Ack1 UBA domain increases the stability of EGFR and leads to prolonged Ras/Raf/MAPK activation (30). The UBA domain of Tnk1 may play a similar role in regulating signaling by associated receptor tyrosine kinases.

Previous studies have suggested both pro-oncogenic and pro-apoptotic/tumor suppressive functions for Tnk1 (2, 11, 12, 14, 15). Our data shed light on the mechanism of Tnk1 activation in the Hodgkin’s lymphoma cell line L540 (15). Tnk1^Fusion^ is oncogenic in Ba/F3 cells. The importance of the Tnk1 C-terminal region in cellular localization and activity has also been highlighted by recent studies of 14-3-3 interaction (7). On the other hand, Tnk1 induces apoptosis in intestinal epithelia, and expression of Tnk1 is increased in patients with Crohn’s disease (31). Whether the C-terminal region of Tnk1 is involved in these effects, or whether mislocalization contributes to human disease, is currently unknown. The list of known Tnk1 substrates and interacting partners is rather limited and includes Grb2, PLCγ-1, AKT, STAT1, STAT3, and STAT5. The seemingly opposing functions of Tnk1 will be better understood when specific substrates are identified that contribute to downstream signaling in oncogenic and pro-apoptotic cellular contexts.

## Experimental procedures

### Materials

The UniProt IDs for human Tnk1 (isoform 2) and human Ack1 (isoform 1) are Q13470-2 and Q07912, respectively. HeLa and HEK293T cells were purchased from ATCC. Radiolabeled ^32^P-ATP was from PerkinElmer (NEG002A100UC). The following antibodies were used: phospho-STAT3 (CST9145P), STAT3 (CST4904S), phospho-AKT (CST4060S), AKT (CST9272S), FLAG (SigmaA8592), SBP (MilliporeMAB10764), Tnk1 (sc-390359), Tnk1 pY277 (MilliporeABS80), pTyr (Millipore05-321), GFP (sc-9996), His-tag (Biolegend906101), GST (MolecularProbes 83C1-3), PKM (sc-365684), Lamin A/C (sc-20681). *(R)*-9b inhibitor was purchased from Millipore Sigma (SML2073). Poly(Glu, Tyr) was purchased from Millipore Sigma (P0275). The peptides were synthesized and purified as described previously (21).

### DNA constructs

pDONR223-TNK1 was a gift from William Hahn & David Root (Addgene plasmid # 23894). For HEK 293T expression of N-terminal FLAG-tagged Tnk1 (UniProtKB - Q13470-2), the Tnk1 coding sequence was amplified by PCR and subcloned into p3xFLAG-CMV-7.1 (Sigma) using EcoRI and BamHI restriction sites. Tnk1 mutants were generated by site-directed mutagenesis using the Agilent QuikChange II kit. For expression in HeLa cells, Tnk1 DNAs were subcloned into pEGFP-C1 (BD Biosciences Clontech) using EcoRI and BamHI restriction sites. For baculoviral expression of Tnk1^ΔCT^ and Tnk1^ΔSAMΔCT^ in Sf9 cells, we first subcloned the DNA sequence encoding a GST-tag and PreScission cleavage site sequence from the pGEX-6p-1 (Millipore Sigma) into the baculoviral expression vector pFastBacHTa (ThermoFisher) using EcoRI and NotI restriction sites. We used PCR to amplify the sequences encoding Tnk1^ΔCT^ (residues 1–510) and Tnk1^ΔSAMΔCT^ (residues 111–510) and ligated them into the modified pFastBacHTa vector backbone using XbaI and KpnI restriction sites.

### Cell culture and transfections

All mammalian cells were cultured in Dulbecco’s Modified Eagle Medium (DMEM) at 4.5 g/l supplemented with 10% fetal bovine serum (FBS) and 1× penicillin and streptomycin. HEK 293T cells were seeded between 2 × 10^6^–4 × 10^6^ per 10 cm dish and left overnight in DMEM supplemented with 10% FBS and 1× penicillin and streptomycin. Cells were transfected according to the TransIT (Mirus) protocol with 5 to 15 μg of DNA at a ratio of 3 μl per 1 μg of DNA. HeLa cells were seeded between 2 × 10^6^–4 × 10^6^ per 10 cm dish and left overnight in DMEM supplemented with 10% FBS and 1× penicillin and streptomycin. Cultures for fluorescence microscopy were seeded at 0.5 × 10^6^–1 × 10^6^ per well of a 6-well dish onto a coverslip. Cells were transfected according to the Lipofectamine 3000 (ThermoFisher) protocol with 10 to 15 μg of DNA per 10 cm dish and 2.5-5 μg of DNA per well of a 6-well dish.

### Mammalian cell lysis

Cells seeded onto 10 cm dishes were harvested in 1× PBS using a cell scraper and transferred to a microcentrifuge tube. Cells were pelleted *via* centrifugation at 7,500*g* at 4 °C for 5 min. Pellets were resuspended in lysis buffer (25 mM Tris pH 7.5, 1 mM EDTA, 100 mM NaCl, 1% Triton X-100, 2 mM PMSF, 0.1 mM sodium orthovanadate, 5 μg/ml aprotinin, 5 μg/ml leupeptin) and left on a rotator for 1 h at 4 °C. Cell suspension was then centrifuged at 15,000*g* at 4 °C for 5 min to isolate the cell debris from the soluble lysate. Protein concentrations were measured using a colorimetric Bradford protein assay. Lysates were then used for Western blot analysis or immunoprecipitation.

### Immunoprecipitation

FLAG-tagged Tnk1 proteins (1 mg) were immunoprecipitated by incubating cell lysate with 20 μl Anti-FLAG affinity resin beads (MilliporeSigma) on a rocker for 2 h at 4 °C. The beads were washed five times by resuspending in 1 ml mammalian cell lysis buffer and either subsequently used for the radioactive kinase activity assay or boiled in 5× Laemmli buffer for Western blot analysis.

### Baculoviral expression in Sf9 cells and protein purification

Sf9 cells (600 ml at 1.8 × 10^6^ cells/ml) were cultured in suspension in Sf-900 medium (Gibco) supplemented with 2.5% FBS and 0.5× penicillin and streptomycin. Expression of Tnk1^ΔCT^ and Tnk1^ΔSAMΔCT^ in Sf9 cells was carried out using the Bac-to-Bac Baculovirus Expression System (Invitrogen). For protein purification, one Sf9 pellet was resuspended in 30 ml of lysis buffer (50 mM Tris pH 8, 100 mM NaCl, 1% NP-40, 10 mM imidazole, 2 mM PMSF, 0.1 mM sodium orthovanadate, 10 μg/ml aprotinin, and 10 μg/ml leupeptin). The cell suspension was lysed twice in a French Pressure cell. The mixture was centrifuged at 40,000*g* at 4 °C for 30 min to pellet cell debris. The soluble fraction was filtered twice using a 5 μM syringe filter followed by a 0.8 μM syringe filter. Cleared lysate was incubated with 5 ml of Ni-NTA resin for 1 h at 4 °C. The resin was washed on a gravity column with 200 ml wash buffer A (20 mM Tris pH 8, 500 mM NaCl, 25 mM imidazole, 10% glycerol), followed by 10 ml of wash buffer B (20 mM Tris pH 8, 10% glycerol). The His-tagged protein was eluted by a series of steps with increasing imidazole concentrations (75 mM, 100 mM, 125 mM, and 150 mM) in 20 mM Tris pH 8, 10% glycerol. One milliliter fractions were collected and analyzed by SDS-PAGE and Coomassie staining.

### *In Vitro* kinase activity assay

The activity assay was performed using either immunoprecipitated FLAG-tagged Tnk1 proteins or purified Tnk1^ΔCT^ from Sf9 cells. Reactions contained 30 mM Tris (pH 7.5), 20 mM MgCl_2_, 1 mg/ml bovine serum albumin, 1 mM sodium orthovanadate, 100 to 500 cpm/pmol [γ^32^P] ATP, and varying concentrations of peptide substrate and unlabeled ATP as described in the figure legends. Assays were carried out at 30 °C and were stopped by addition of cold 10% trichloroacetic acid. Samples were vortexed and then centrifuged at 10,000*g* for 2 min. Supernatants were spotted onto 2 × 2 cm pieces of Whatman P81 paper. The P81 papers were then washed for 10 min by gently rocking in 0.5% phosphoric acid for a total of three washes. After a final acetone wash, the P81 papers were air dried, and ^32^P incorporation was quantified using a scintillation counter.

### Enzyme kinetics

Values of K_m_ for ATP and WASP peptide substrate for Tnk1 were determined using the phosphocellulose paper assay and [γ^32^-P ATP]. Reactions contained 500 nM purified Tnk1 and varying concentrations of ATP or WASP substrate. For each concentration of substrate or ATP, reactions were stopped at 2, 4, 6, 8, and 10 min, and initial rates were calculated using the slopes of the cpm *versus* time plots. Values for K_m_ and V_max_ were determined by nonlinear fitting of the rate *versus* [peptide] and rate *versus* [ATP] plots using GraphPad Prism v6.

### Inhibitor screen and IC_50_ measurement

Inhibitor screens were carried out using the phosphocellulose paper assay and [γ^32^-P ATP]. Tnk1 was pre-incubated with each inhibitor for 10 min prior to the start of the reactions. Reactions contained 500 nM purified Tnk1, 10 μM inhibitor, 1 mM WASP substrate, 1 mM ATP, and a final concentration of 1% dimethyl sulfoxide. The IC_50_ of *(R)*-9b was determined using varying inhibitor concentrations, and the data were analyzed using GraphPad Prism v6.

### Fluorescence microscopy and quantification

HeLa cells seeded onto coverslips were transfected with Lipofectamine 3000. Samples were fixed in 1% paraformaldehyde in 1× PBS and subsequently stained using wheat germ agglutinin conjugated to rhodamine (Vector Laboratories, RL-1022). Coverslips were then mounted onto slides using VectaShield mounting media containing DAPI. Cells were washed three times with 1× PBS between each step. For quantifying microscopy images with ImageJ, at least 20 cells per condition were analyzed using a fixed region of interest for measuring the mean pixel density of both the nucleus and the cytoplasm. Three regions of interest were measured per subcellular region of interest and subtracted by the background pixel density.

### Cell fractionation

HeLa cells transfected with GFP Tnk1 proteins were subsequently fractionated with the Subcellular Protein Fractionation Kit for Cultured Cells (ThermoFisher, 78840) using the manufacturer’s protocol. Fractionations were carried out using approximately five million cells per condition.

## Data availability

All data and supporting information are contained within this article.

## Supporting information

This article contains supporting information (32).

## Conflict of interest

The authors declare that they have no conflicts of interest with the contents of this article.
