# Supplementary material for: The noncatalytic regions of the tyrosine kinase Tnk1 are important for activity and substrate specificity
Source: J Biol Chem. 2022 Nov 2;298(12):102664. doi: 10.1016/j.jbc.2022.102664 (PMC9703632; doi:10.1016/j.jbc.2022.102664)
Supplement: Supporting information [file mmc1.pdf]

Supporting Information for

The noncatalytic regions of the tyrosine kinase Tnk1 are important for activity and substrate specificity

Sultan Ahmed[1] and W. Todd Miller[1*, 2]

[1]: *Department of Physiology and Biophysics, Stony Brook University, Stony Brook, NY 11794, United States*

[2]: *Department of Veterans Affairs Medical Center, Northport, NY 11768*

*Email: todd.miller@stonybrook.edu

List of materials included: Supplementary figures 1-6.

A

```
Abl1 248 LGGGQYGEVYEGVW---KKYSLTVAVKTLK---EDTM--EVEEFLKEAAVMKEIKHPNLV
Tnk1 122 LGSGCFGVVHRGLWTLPSGKSVPVAVKSLRVGPEGPMGTELGDFLREVSVMMNLEHPHVL
         ** *  * *  * *      *  **** *    *  *  *   ** *  **    **

Abl1 300 QLLGVCTREPPFYIITEFMTYGNLLDYLRECNRQEVNAVVLLYM-ATQISSAMEYLEKKN
Tnk1 182 RLHGLVLGQP-LQMVMELAPLGSLHARLTAPAPTPPLLVALLCLFLRQLAGAMAYLGARG
          * *     *      *    * *   *          * **     *   ** **

Abl1 359 FIHRDLAARNCLVGENHLVKVADFGLSRLMTG--DTYTAHAGAKFPIKWTAPESLAYNKF
Tnk1 241 LVHRDLATRNLLLASPRTIKVADFGLVRPLGGARGRYVMGGPRPIPYAWCAPESLRHGAF
           ***** ** *       ******* *   *    *        *  * *****    *

Abl1 417 SIKSDVWAFGVLLWEIATYGMSPYPGIDLSQVYELLEKDYRMERPEGCPEKVYELMRACW
Tnk1 301 SSASDVWMFGVTLWEMFSGGEEPWAGVPPYLILQRLEDRARLPRPPLCSRALYSLALRCW
         *  **** *** ***    *  *  *         **   *  **  *    * *   **

Abl1 477 QWNPSDRPSFAEI
Tnk1 361 APHPADRPSFSHL
            * *****
```

B

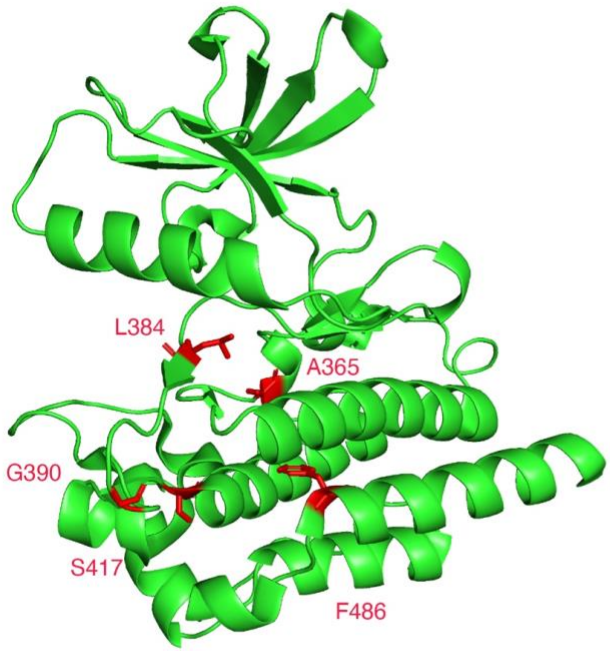

Supplemental Figure 1. Abl-Tnk1 homology. (A) The sequences of the kinase domains of human Abl1 (Uniprot P00519, residues 248-489) and Tnk1 (Uniprot Q13470, residues 122-373) were aligned using the SIM alignment tool (web.expasy.org). Conserved residues are indicated by asterisks below the sequences. Positions that are potential determinants of specificity (specificity score ≥ 0.675; Creixell et al., Cell 2015, 163: 187-201) are indicated with red arrowheads. (B) Crystal structure of the Abl kinase domain (pdb 1m52) with the conserved residues shown in red in stick format. The figure was prepared using Pymol.

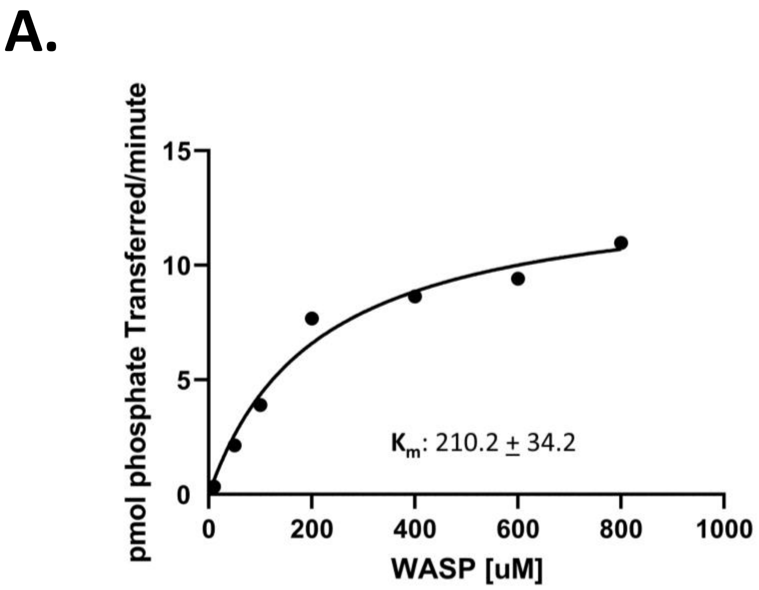

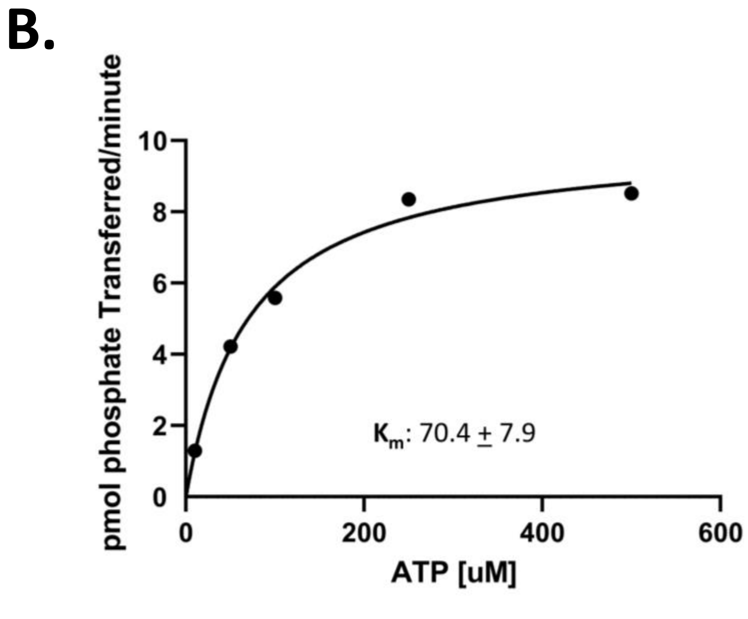

Supplemental Figure 2: Kinetic analyses of Tnk1$^{\Delta CT}$. A) Activity assays were carried out with 1 mM ATP and varying concentrations of the WASP peptide substrate. B) Activity assays were carried out with 1 mM WASP peptide and varying concentrations of ATP. Reactions contained 500nM enzyme.

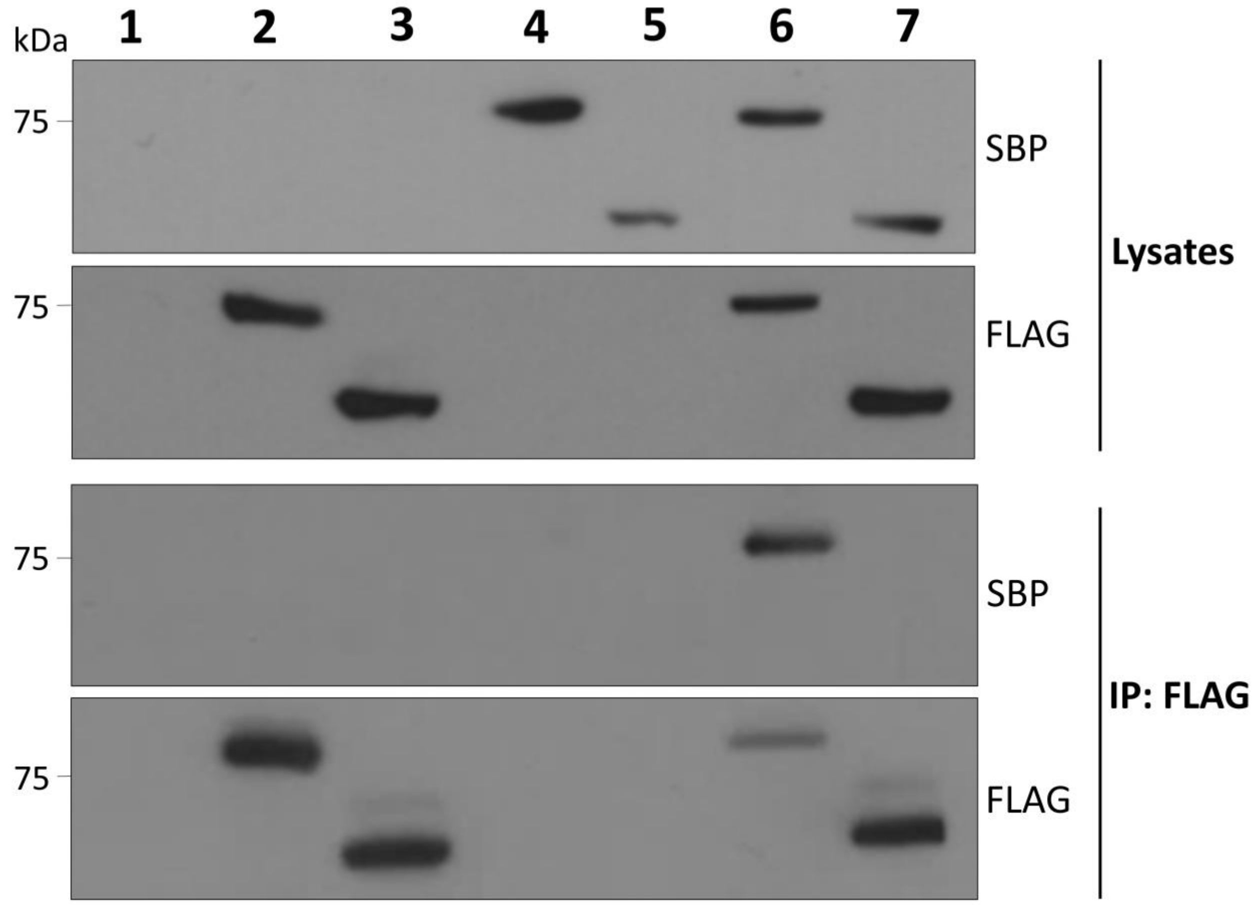

Supplemental Figure 3: Expression and Immunoprecipitation of FLAG-tagged and SBP-tagged Tnk1^{WT} and Tnk1$^{\Delta SAM}$. HEK293T cells were singly transfected or co-transfected as follows: 1) Untransfected HEK293T cells 2) FLAG-tagged Tnk1^{WT} 3) FLAG-tagged Tnk1$^{\Delta SAM}$ 4) SBP-tagged Tnk1^{WT} 5) SBP-tagged Tnk1$^{\Delta SAM}$ 6) Co-transfection of FLAG-tagged Tnk1^{WT} and SBP-tagged Tnk1^{WT} 7) Co-transfection of FLAG-tagged Tnk1$^{\Delta SAM}$ and SBP-tagged Tnk1$^{\Delta SAM}$. Top: Lysates were probed with anti-FLAG and anti-SBP antibodies. Bottom: The lysates were subjected to immunoprecipitation with anti-FLAG resin. The anti-FLAG precipitates were analyzed by Western blotting. The two right-hand lanes of these gels, showing results from the doubly transfected cells, are shown in Fig. 3A. This experiment has been repeated four times with similar results.

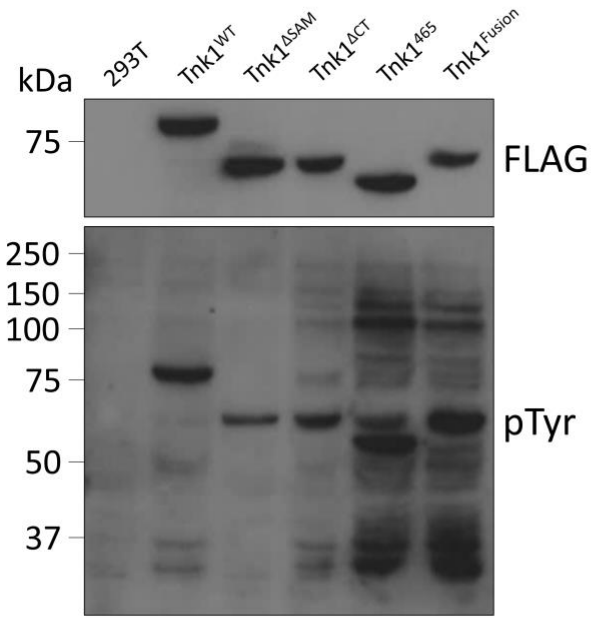

Supplemental Figure 4: Cellular activity of all Tnk1 mutants. Lysates of HEK 293T cells expressing FLAG-tagged Tnk1^{WT} and mutants were analyzed by anti-FLAG and anti-pTyr Western blotting.

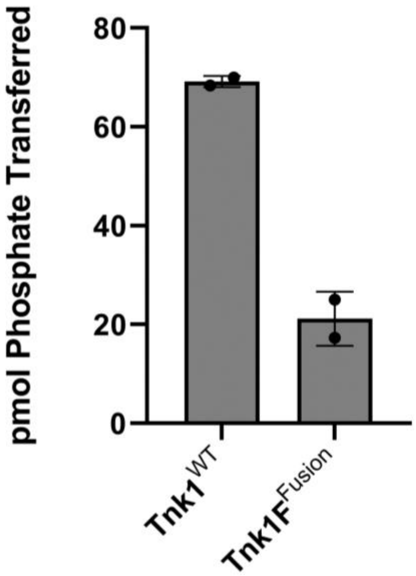

Supplemental Figure 5: Activity of Tnk1^{WT} and Tnk1^{Fusion} Using poly(Glu, Tyr) Substrate. The *in vitro* activity of Tnk1^{WT} and Tnk1^{Fusion} was measured against poly(Glu, Tyr) (1mg/ml) using the phosphocellulose binding assay and γ^{32}-ATP.

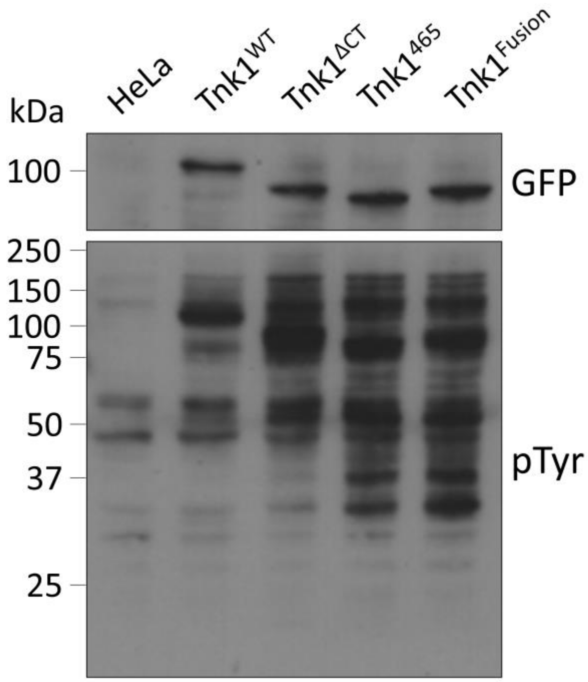

Supplemental Figure 6: Activity of Tnk1 Mutants in HeLa Cells. Lysates from HeLa cells expressing Tnk1^{WT} and mutants were analyzed by anti-GFP and anti-pTyr Western blotting.
